# Analysis of reproduction-related transcriptomes on pineal-hypothalamic-pituitary-ovarian tissues during estrus and anestrus in Tan sheep

**DOI:** 10.3389/fvets.2022.1068882

**Published:** 2022-11-24

**Authors:** Shihao Wei, Xiaolong Kang, Chaoyun Yang, Feng Wang, Tianshu Dai, Xingru Guo, Ziming Ma, Chenglong Li, Hongxi Zhao, Xingang Dan

**Affiliations:** Key Laboratory of Ruminant Molecular and Cellular Breeding, School of Agriculture, Ningxia University, Yinchuan, China

**Keywords:** Tan sheep, seasonal reproduction, transcriptome, PHPO axis, estrus, anestrus

## Abstract

Seasonal estrus is an important factor limiting the fertility of some animals such as sheep. Promoting estrus in the anestrus season is one of the major ways in improving the fecundity of seasonally breeding animals. The pineal-hypothalamus-pituitary-ovary (PHPO) axis plays a decisive role in regulating animal reproduction. However, the molecular mechanisms by which the PHPO axis regulates seasonal reproduction in animals are not well understood, especially in Tan sheep. To this end, we collected pineal, hypothalamus, pituitary and ovary tissues from Tan sheep during estrus and anestrus for RNA-Sequencing, and performed bioinformatics analysis on the entire regulatory axis of the pineal-hypothalamic-pituitary-ovary (PHPO). The results showed that 940, 1,638, 750, and 971 DEGs (differentially expressed genes, DEGs) were identified in pineal, hypothalamus, pituitary and ovary, respectively. GO analysis showed that DEGs from PHPO axis-related tissues were mainly enriched in “biological processes” such as transmembrane transport, peptide and amide biosynthesis and DNA synthesis. Meanwhile, KEGG enrichment analysis showed that the bile acid secretion pathway and the neuroactive ligand-receptor interaction pathway were significantly enriched. Additionally, four potential candidate genes related to seasonal reproduction *(VEGFA, CDC20, ASPM*, and *PLCG2*) were identified by gene expression profiling and protein-protein interaction (PPI) analysis. These findings will contribute to be better understanding of seasonal reproduction regulation in Tan sheep and will serve as a useful reference for molecular breeding of high fertility Tan sheep.

## Introduction

Some animals in nature undergo some behavioral changes with the change of seasons, such as fish migration, turtle hibernation, and bird migration. It has been found that animal reproduction activity varies with the seasons, which is not only related to the ambient temperature and light, but also closely related to the endogenous regulation of animals themselves. Studies have shown that genes involved in the regulation of animal reproduction change rhythmically with the seasons and play an important role in the regulation of seasonal reproduction (1). The reproductive activity of seasonal breeding animals is governed by a regulatory network of the Pineal—Hypothalamus—Pituitary—Ovary (PHPO) axis. The mammalian pineal is a neuroendocrine sensor whose primary function is to convert photoperiodic information into nocturnal hormonal signals for melatonin synthesis and secretion (2). The hormonal activity of the pineal is influenced by the light and dark cycles, as well as seasonal cycle, and it plays an important role in the seasonal reproductive shift of animals (3). The hypothalamus, an important part of the central nervous system, senses changes in the light and dark signals from the external environment *via* the retina-supraoptic nucleus bundle, synchronizing the biological rhythms with environmental light and dark changes (4). The pituitary, an important reproductive endocrine regulatory organ in animals, secretes adrenocorticotropic hormone, thyroid stimulating hormone, luteinizing hormone, follicle stimulating hormone, growth hormone and prolactin (5). Among them, LH and FSH play a key role in the seasonal breeding of animals. Furthermore, the pituitary gland is not only controlled by the hypothalamus, but also influences ovarian function through follicle stimulating hormone and luteinizing hormone, and it serves as a link in the hypothalamic-pituitary-ovarian (HPO) axis. The ovary is an important reproductive organ in female mammals and its function is regulated by the HPO axis (6). Moreover, the ovary can regulate the synthesis and secretion of reproduction-related hormones in the hypothalamus and pituitary *via* positive and negative feedback mechanisms (7).

In the past, studies have found a number of important genes and pathways regulating animal reproduction by high-throughput sequencing in the HPO axis of female animals (8, 9). In sheep, many studies have focused on transcriptome sequencing analysis of muscle tissue (10), adipose tissue (11) or skin tissue (12) and have identified a number of candidate markers. Meanwhile, RNA-Seq was also been used to investigating reproductive regulation mechanism of animals. Ullah et al. (13) have discovered six miRNAs associated with estrus including miR-143 and miR-199a after transcriptome sequencing analysis of the pituitary of sheep during estrus and anestrus. Also, Di et al. (14) used transcriptome sequencing to screen for differentially expressed miRNAs between the pineal of estrus and anestrus sheep and showed that miR-89 negatively regulates the expression of mRNA involved in the melatonin synthesis rate-limiting enzyme arylalkylamine N-acetyltransferase, which in turn regulates reproduction in sheep. None of these studies, however, examined the pineal along with the hypothalamus, pituitary and ovary, which might have missed some novel findings in the regulation of seasonal reproduction in animals. In this study, mRNA from the pineal, hypothalamus, pituitary, and ovarian tissues of female Tan sheep during estrus and anestrus were first sequenced separately. These sequencing results were then analyzed by bioinformatics method to uncover the key genes and signal pathways that regulate seasonal reproduction in the entire PHPO axis of Tan sheep.

## Materials and methods

### Experimental animals and sample collection

All methods of this study were performed in accordance with the Regulations for the Administration of Laboratory Animal Affairs (revised, 2017) providing animal experiments and animal care. All experimental animal studies were reviewed and approved by the Animal Welfare Committee of Ningxia University (permit number NXUC20180306).

In this study, 12 Tan sheep (14 months old) in similar body condition during the anestrus season (early April) were selected and kept individually for 1 week at a Tan sheep breeding farm in Ningxia Autonomous Region, and three female Tan sheep were proved not to be in heat by test rams again. Subsequently, frozen tubes of pineal, hypothalamus, pituitary and ovarian tissues were collected from each of the three Tan sheep after humanitarian execution at the slaughterhouse and immediately cast into liquid nitrogen for backup until RNA extraction. At the same time, three female Tan sheep were screened from the remaining nine Tan sheep during the same year's anestrus season (late August, 18 months old) by means of a test ram, and the pineal, hypothalamus, pituitary gland and ovary tissues were collected in frozen tubes after slaughter at the slaughterhouse, and immediately placed in liquid nitrogen until RNA extraction.

### RNA extraction and library construction and sequencing

Total RNA was extracted from all samples according to the instructions of TRIZOL RNA Extraction Kit (Invitrogen, Carlsbad, CA, USA). The integrity and quality of sample RNA were assessed by the Agilent 2,100 bio analyze system (Agilent Technologies, Santa Clara, CA, USA), and samples RNA that passed the test were used for subsequent library construction.

The NEBNext^®^ Ultra™ RNA Library Prep Kit was used for library construction. mRNAs with polyA tails was enriched by Oligo (dT) magnetic beads and resulting RNA was subsequently randomly interrupted with divalent cations in NEB Fragmentation Buffer. The fragmented mRNA was used as a template, and random oligonucleotides were used as primers to synthesize the first strand of cDNA in M-MuLV reverse transcriptase system, followed by RNaseH degradation of RNA strands and dNTP synthesis in DNA polymerase I system. The purified double-stranded cDNAs were end-repaired, A-tailed and connected to sequencing adapters, and the cDNAs of about 200 bp were screened for PCR amplification using AMPure XP beads. After that, the PCR products were purified again using AMPure XP beads to finally obtain the library.

After constructing the library, it was quantified with a Qubit 2.0 Fluorometer, diluted to 1.5 ng/μl, and tested for the insert size with an Agilent 2,100 bioanalyzer. Subsequently, qRT-PCR was used to accurately quantify the effective concentration of the library (above 2 nM) to ensure the library's quality.

### Comparison of data processing and reference genome analysis

The sequenced fragments were converted into fastq format reads by CASAVA base identification of the images from the high-throughput sequencer. After obtaining the raw data to ensure quality and reliability of the data, Trim-galore software (version 0.6.6, https://www.bioinformatics.babraham.ac.uk/projects/trim_galore/) was used to remove N-containing reads, filter reads with adapters and remove low-quality reads. To obtain the comparison efficiency of each sample reads and the position of reads on the genome, all trimmed data were mapped to the sheep reference genome *Ovis aries* (ftp:/ftp.ncbi.nlm.nih.gov/genomes/all/GCA/000/298/735/ GCA000298735.2Oarv4.0).

### Differentially expressed genes identification and GO/KEGG enrichment analysis

Gene expression levels were quantified using the feature Counts (version 1.5.0-p3, http://subread.sourceforge.net/) tool in subread software, and gene expression values for RNA-seq were calculated using FPKM. DEGs were detected using DESeq2 (version1.24.0, http://www.bioconductor.org/packages/release/bioc/html/DESeq2.html) with differential gene screening criteria of |log2(FoldChange)| ≥ 3 and padj ≤ 0.01. GO and KEGG analyzes were performed on the DAVID (version 2021, https://david.ncifcrf.gov/) website.

### Trend analysis of gene expression

The STEM (Short Time-series Expression Miner, version 1.3.13, http://www.sb.cs.cmu.edu/stem) analysis program was used to identify significant gene expression profiles and to compare and visualize clusters of genes with similar expression trends in estrus and anestrus. Each gene was assigned to the closest profile using a Pearson correlation-based distance metric. To determine the significance level of the clustering profiles, alignment-based tests were used to quantify the expected number of genes that would be assigned to each profile. The gene expression levels used for analysis are expressed as FPKM values, with a *P* value < 0.05 in the clustering profile considered significantly enriched. Where the standardization method used for STEM analysis was chosen as “Log normalize data” and the number of trends for analysis using the “TEM Clustering Method” was chosen as “50,” other parameters were selected as default values.

### PPI analysis of differentially expressed MRNAs

To elucidate the potential relationships between DEGs and different tissues over time, we analyzed colored Clusters using STEM. DEGs with significant differences and similar trends contained in colored clusters (25, 24, 8, 35) were then subjected to protein-protein interaction (PPI) analysis in the STRING database (version 11.5, https://string-db.org/). Cluster 25, 24, 8, and 35 were examined with confidence criteria of 0.7, 0.92, 0.5, and 0.9, respectively. The Cytoscape tool (version 3.9.0, https://cytoscape.org/) was used to further visualize the interaction networks. The CytoHubba plugin's network topology analysis approach (Degree) was used to determine the core genes.

### RT-QPCR validation of DEGs

RNA was extracted from four tissue samples of six Tan sheep stored in liquid nitrogen, and after determination of satisfactory concentration and purity, diluted to 1 μg-μl^−1^ with double-distilled water. First strand of cDNA was prepared using the PrimeScript™ RT reagent Kit with gDNA Eraser (TaKaRa, Perfect Real Time, Cat#RR047A) kit. For each tissue, six DEGs were randomly chosen to design unique primers, with the β-actin gene serving as an internal reference gene (Supplementary Table S1). RT-qPCR analysis of DEGs from four Tan sheep tissue was performed with the TB Green^®^ Premix Ex Taq™ II (Takara, Tli RNaseH Plus, Cat#RR820A) kit. The reaction system was: 2 × TB Green Premix Ex Taq II 10 μl, Primer F 0.8 μl, Primer R 0.8 μl, RNase Free ddH2O 6.4 μl, cDNA template 2 μl; reaction conditions: pre-denaturation at 95 °C for 30 s, denaturation at 95 °C for 10 s, annealing at 60 °C 30 s, total 40 cycles. Three replicates of each sample were used to calculate the relative expression by the 2^−ΔΔCt^ method. In order to confirm the accuracy of the RNA-Seq results, their results of RT-qPCR were compared with those of RNA-Seq.

### Statistical analysis

Statistical processing of RT-qPCR results was performed using SPSS (version 19.0, International Business Machines Corporation). Student's *t*-test for independent samples was used to test for differences between the two groups. The differences were statistically significant (*P* < 0.05). Data are expressed as mean ± standard deviation.

## Results

### Quality analysis of RNA sequencing data

The average number of reads for raw and clean reads was over 76 million and 73.5 million, respectively. After alignment with the *Ovis aries* genome, an average alignment rate of 89.49% was obtained for all samples, with a minimum value of 88.52% and a maximum value of 92.31% (Supplementary Table S2). Therefore, the quality of data can meet the requirements for the subsequent analysis of DEGs between the estrus and anestrus groups of Tan sheep.

### Identification of differentially expressed genes

**Figure 1:**
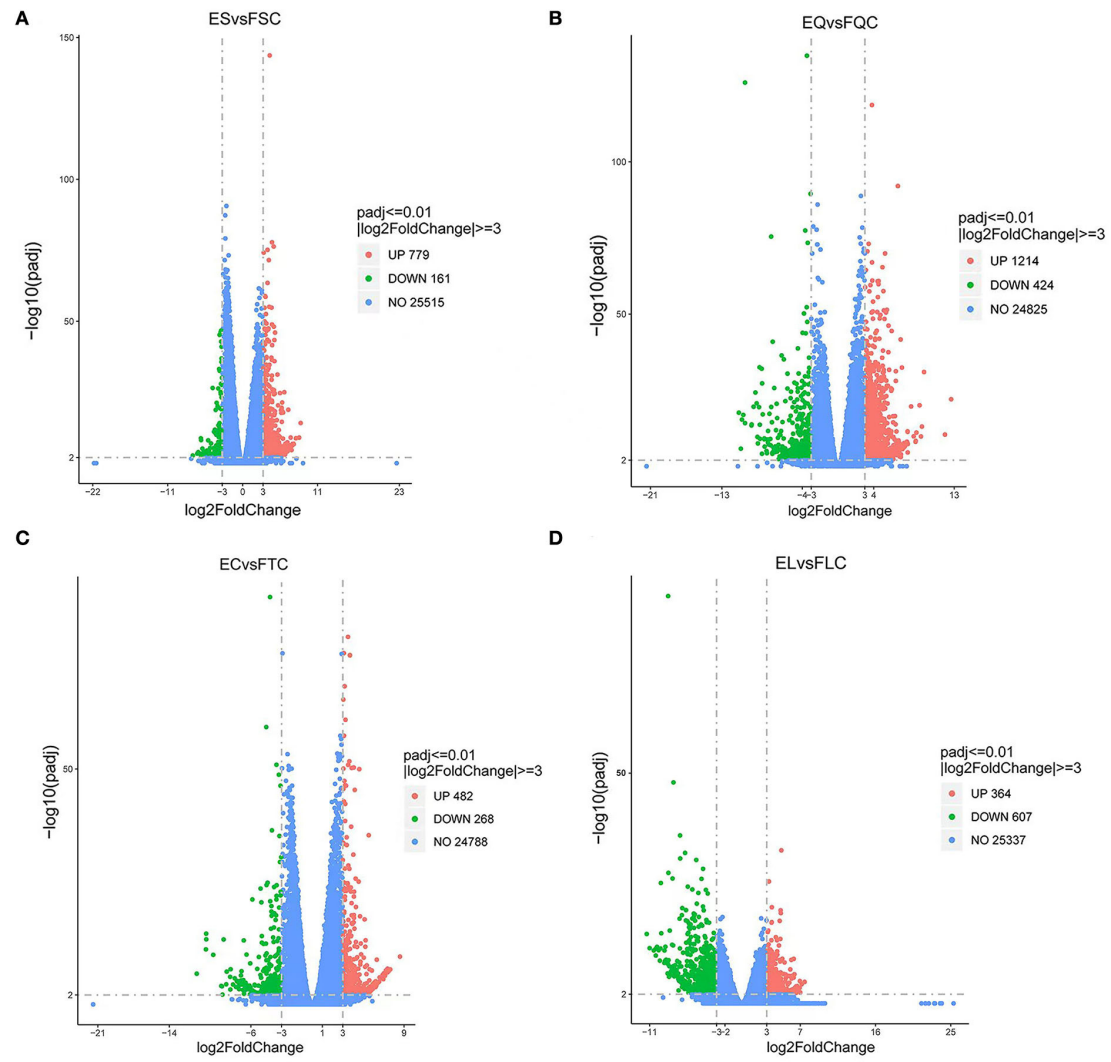
Volcano map of differentially expressed genes. **(A)** Pineal, **(B)** Hypothalamus, **(C)** Pituitary, and **(D)** Ovary map of differentially expressed genes in the ovary. Blue dots indicate genes that did not show significant changes between estrus group (EG) and anestrus group (AG) samples. Green dots represent down-regulated genes and red dots represent up-regulated genes.

**Figure 2:**
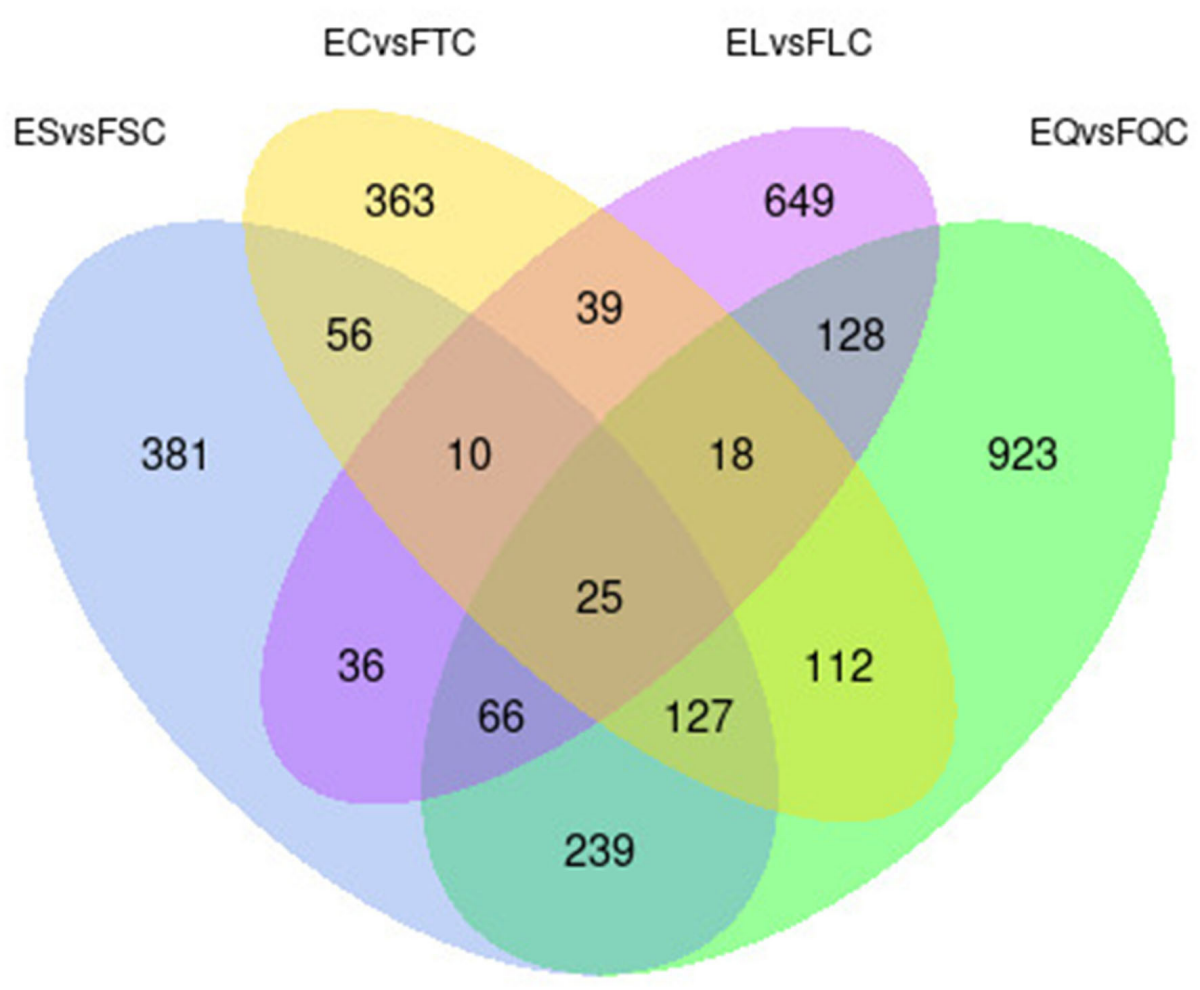
Venn diagram of differentially expressed genes in four tissues.

**Figure 3:**
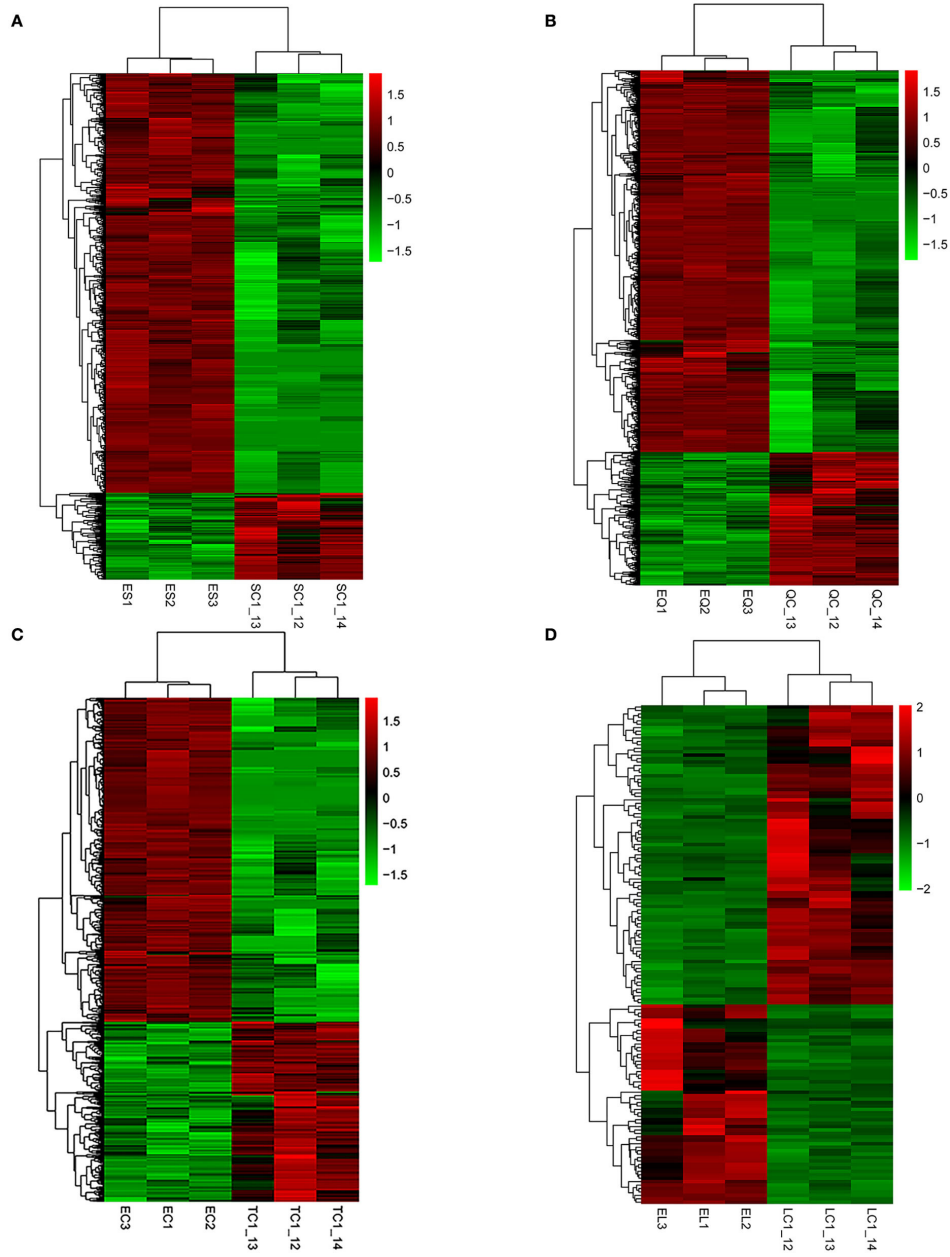
Hierarchical clustering analysis of differentially expressed genes. **(A–D)** Represent the pineal, hypothalamus, pituitary and ovarian tissue, respectively. The colored bars indicate the expression level. Green represents genes with low expression levels and red represents genes with high expression.

To investigate DEGs in four tissues of Tan sheep during estrus and anestrus, gene expression levels were quantified by FPKM in the pineal, hypothalamus, pituitary and ovary. A total of 940 (up-regulated 779, down-regulated 161), 1,638 (up-regulated 1,214, down-regulated 424), 750 (up-regulated 482, down-regulated 268), and 971 (up-regulated 364, down-regulated 607) DEGs were found in the pineal, hypothalamus, pituitary and ovary tissues in estrus and anestrus periods, respectively (Figure 1, Supplementary Table S3). According to Venn diagram analysis, a total of 25 DEGs were co-expressed in the four tissues, with the number of tissue-specific DEGs being 381, 923, 363, and 649 in the pineal, hypothalamus, pituitary and ovary, respectively. This indicates significant changes in gene transcription on the PHPO axis during the transition between breeding seasons in Tan sheep (Figure 2). In order to examine the expression patterns of DEGs in different samples, hierarchical clustering analysis was employed (Figure 3). The results showed similar expression patterns in the pineal, hypothalamus and pituitary tissues, but distinct expression patterns in the ovarian tissue, which could be explained by the specificity of ovarian tissue and variations in ovarian development and ovulatory cycles in Tan sheep.

### GO entries enriched for DEGs in PHPO axis-related tissues

**Figure 4:**
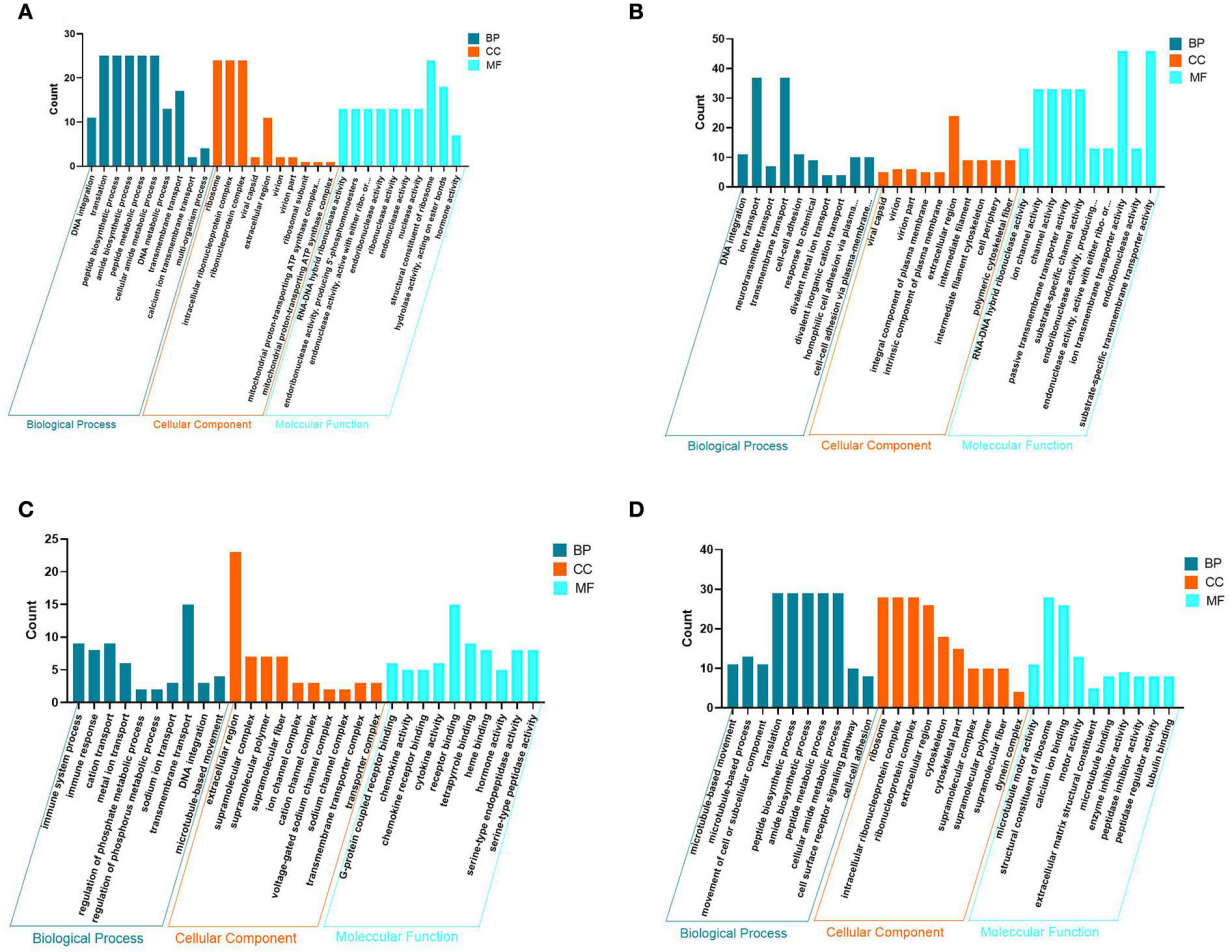
Map of GO entries for differentially expressed genes. **(A–D)** Represent the pineal, hypothalamus, pituitary and ovarian tissue, respectively. Dark blue indicates biological processes; yellow indicates cellular components; light blue indicates molecular functions.

To understand the molecular functions of DEGs at the transcriptional level of the PHPO axis, DEGs were annotated using GO enrichment (Figure 4, Supplementary Table S4). The 940 DEGs on the pineal gland were found to be enriched to 502 GO entries, including 258 biological processes (BP), 63 cellular components (CC), and 181 molecular functions (MF). Processes such as DNA synthesis, translation and metabolism, peptide and amide synthesis and metabolism were predominant on the BP. It is primarily connected to the mitochondrial proton transport ATP synthase complex, riboprotein complex, virion, extracellular area, and other parts in the CC. In MF, it mostly relates to the activities of ribonucleases, endonucleases, endogenous enzyme activity, structural components of ribosomes, hormone action, etc.

There are 1,638 DEGs enriched to 646 GO entries in the hypothalamus, including 325 BP, 84 CC and 237 MF. The enrichment of DEGs on the hypothalamus is associated with processes such as transport of neurotransmitters, ions and other substances, cell adhesion and other processes. on the CC, there are mainly components of the virion, shell, intrinsic and intrinsic components of the plasma membrane, extracellular regions, intermediate filaments and intermediate filament cytoskeleton. The MF category is associated with RNA-DNA hybrid ribonuclease activity, endonuclease activity, ion channel activity, substrate specific channel activity, and transmembrane transporter activity.

In the pituitary, 750 DEGs were enriched to 514 GO entries, including 225 BP, 72 CC, and 187 MF. BP was mainly enriched to processes such as immune response, ion transport, DNA synthesis, regulation of phosphate metabolic processes and microtubule movement. It was mainly associated with components of ion and sodium channel transporter complexes, extracellular regions, supramolecular complexes, fibers and polymers On the CC. Additionally, there are some category mainly associated with G-protein coupled receptors, chemokine receptors, tetrapyrrole and heme binding, chemokines, cytokines, hormones and serine-type endopeptidase activity in the MF.

In addition, 971 DEGs were enriched to 561 GO entries, including 259 BP, 89 CC, 213 MF in the ovaries. In BP, it was mainly enriched in processes such as movement of microtubules and cellular or subcellular components, peptide and amide synthesis and metabolism, cell surface receptor signaling pathways, cell-cell adhesion. It was mainly associated with ribosomes, ribonucleoproteins, and Intracellular ribonucleoprotein complexes, extracellular regions, skeletal fractions, supramolecular fibers, complexes and polymers, and other components in the CC. While in MF, DEG category is mainly enriched to molecular functions such as serine-type peptidase, hydrolase endopeptidase and endopeptidase inhibitor and regulator activity, nucleoside-triphosphatase and transmembrane receptor protein kinase activity. As a result, it is clear that DNA synthesis, extracellular regions, enzyme and hormone activities are the most common GO entries in the four tissues of the PHPO axis.

### KEGG pathway analysis of DEGs-enriched in PHPO axis

**Figure 5:**
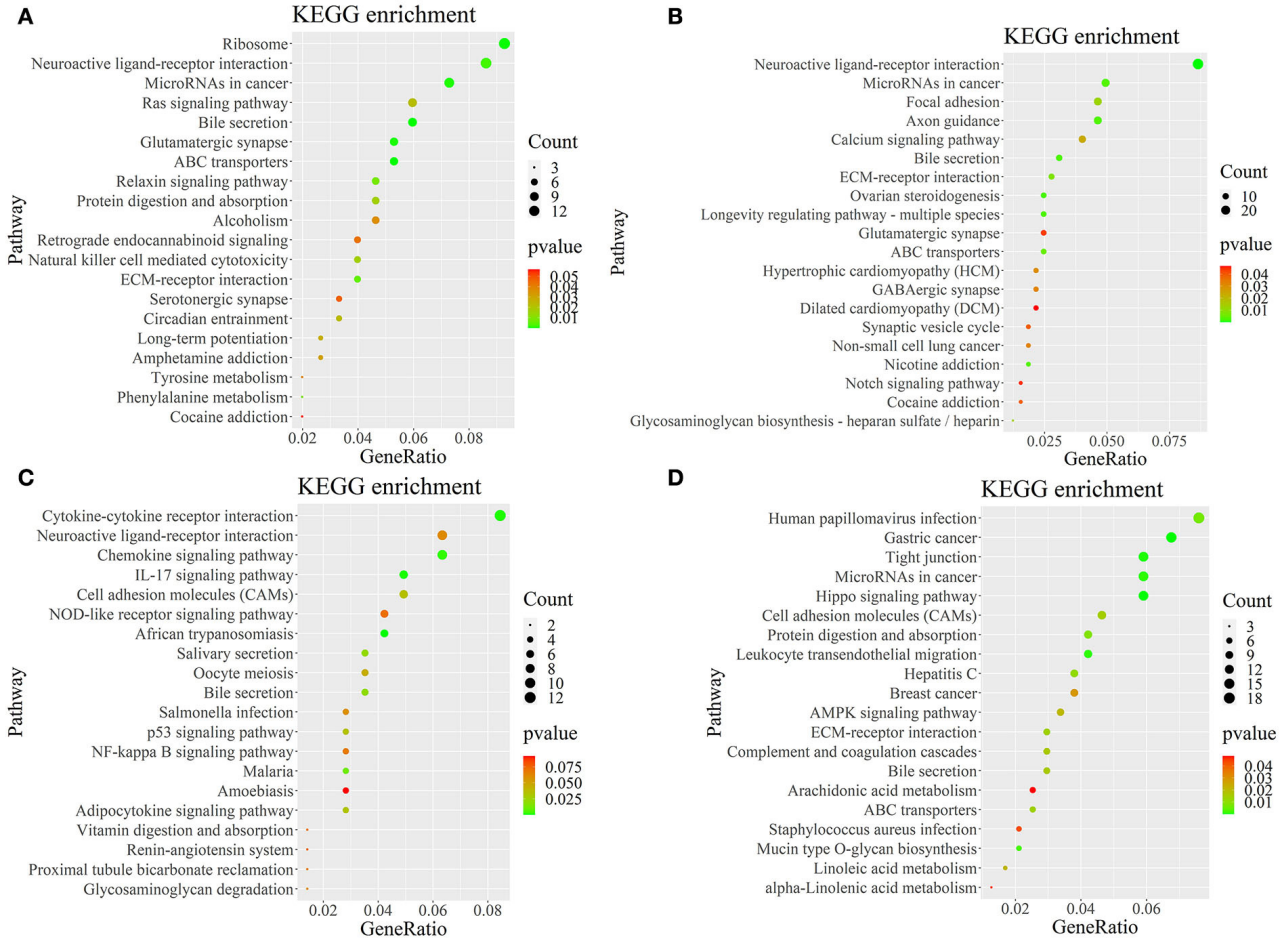
KEGG pathway map of differentially expressed genes. **(A–D)** Represent the pineal, hypothalamus, pituitary and ovarian tissue, respectively. Horizontal coordinates indicate enrichment factors and vertical coordinates indicate GO terms. The color of the dots corresponds to the range of *P*-value, and the size of the dots indicates how many differentially expressed genes are in the pathway.

To further determine the role of specific signaling pathways in the regulation of seasonal reproduction in Tan sheep, we completed an analysis of the KEGG pathway enrichment of DEGs in the PHPO axis of Tan sheep during estrus and anestrus, and the top 20 pathways in the four tissues are displayed in the graphs (Figure 5, Supplementary Table S5). The ribosome signaling pathway had the most DEGs (14) in the pineal gland, and the Ras signaling pathway, relaxin signaling, and circadian regulatory pathways are likely to be important in the regulation of seasonal anestrus in Tan sheep. The neuroactive ligand-receptor pathway has the most DEGs (15) in the hypothalamus, followed by the Notch signaling pathway, ovarian steroidogenesis, and the calcium signaling pathway, which were related to reproduction. In the pituitary, the cytokine-cytokine receptor interaction pathway had the most DEGs (12), whereas the P53 signaling pathway, cell adhesion molecules (CAMs), the NF-kappa B signaling system, and oocyte meiosis were involved in reproduction regulation. In addition, the largest number of DEGs in the ovary was in the human papillomavirus infection pathway (16), and the reproduction-related pathways included the “Hippo” signaling pathway, CAMs, arachidonic acid metabolism, and AMPK signaling pathway. Notably, the bile secretion pathway was the only pathway that was co-enriched in the four tissues during estrus and diestrus (Table 1). The neuroactive ligand-receptor pathway was also found to be co-enriched in pineal, hypothalamic and pituitary tissues (Table 2). Additionally, we also discovered that adenosine triphosphate-binding transporter protein and the ECM-receptor interaction pathway were co-enriched in hypothalamic, pineal and ovarian tissues.

**Table 1:** DEGs significantly enriched in bile secretion pathway.

**Tissues**	**DEGs**
Pineal	ABCB1↑	LOC106991125↑	SLC10A1↑	LOC101121256↑	SLC51A↑
	LOC101112460↑	SLCO1B3↓	LOC105606000↑	LOC105606030↑	
Hypothalamus	SLCO1A2↑	ADCY4↑	SCTR↓	LOC105606000↑	SLC10A1↑
	LOC106991125↑	LOC101112460↑	SLC22A8↓	LOC101121256↑	SLC22A7↑
Pituitary	LOC101108321↓	SLC10A1↑	SLC22A8↓		
	LOC101118398↑	SLC22A1↓			
Ovary	CFTR↓	LOC106991125↑	LOC101112460↑	LOC105606000↑	
	AQP4↓	LOC101110133↑	LOC101121256↑		

“↑” indicates gene up-regulation and “↓” indicates gene down-regulation.

**Table 2:** DEGs significantly enriched in neuroactive ligand-receptor interaction pathway.

**Tissues**	**DEGs**
Pineal	CHRNA2↑	ADRA1A↑	RXFP3↑	GLRA1↑	P2RY4↑	HTR1F↓
	GRM7↑	GRM5↑	GRIN1↑	GRIN2B↑	GALR3↑	LOC101108550↓
	NPY2R↑					
Hypothalamus	P2RX7↑	GRIK3↑	CHRNB3↑	CHRNA2↑	TSHR↓	NMUR2↓
	PRL↓	CHRNA6↑	GRM5↑	GABRD↓	P2RX3↑	RXFP2↓
	GRIN2B↑	GLRA3↑	CHRM1↓	TSHB↓	P2RX2↓	MC3R↓
	GH↓	SCTR↓	CHRNA5↑	GIPR↑	FSHR↑	GABRR2↑
	LHB↓	FSHB↓	CHRNA3↑			
Pituitary	TRHR↑	HCRTR2↓	LOC101121298↓	P2RY4↑	FSHR↑	LOC101121553↓
	GRM7↑	CCKAR↓	GRM4↓			
Ovary	CHRNA2↑	TACR1↓	P2RX2↓	P2RX3↓	CHRNB2↑	GRIN2C↑
	APLNR↑	PTGER3↓	LHCGR↑	NPY5R↑		

“↑” indicates gene up-regulation and “↓” indicates gene down-regulation.

### Analysis of STEM results

**Figure 6:**
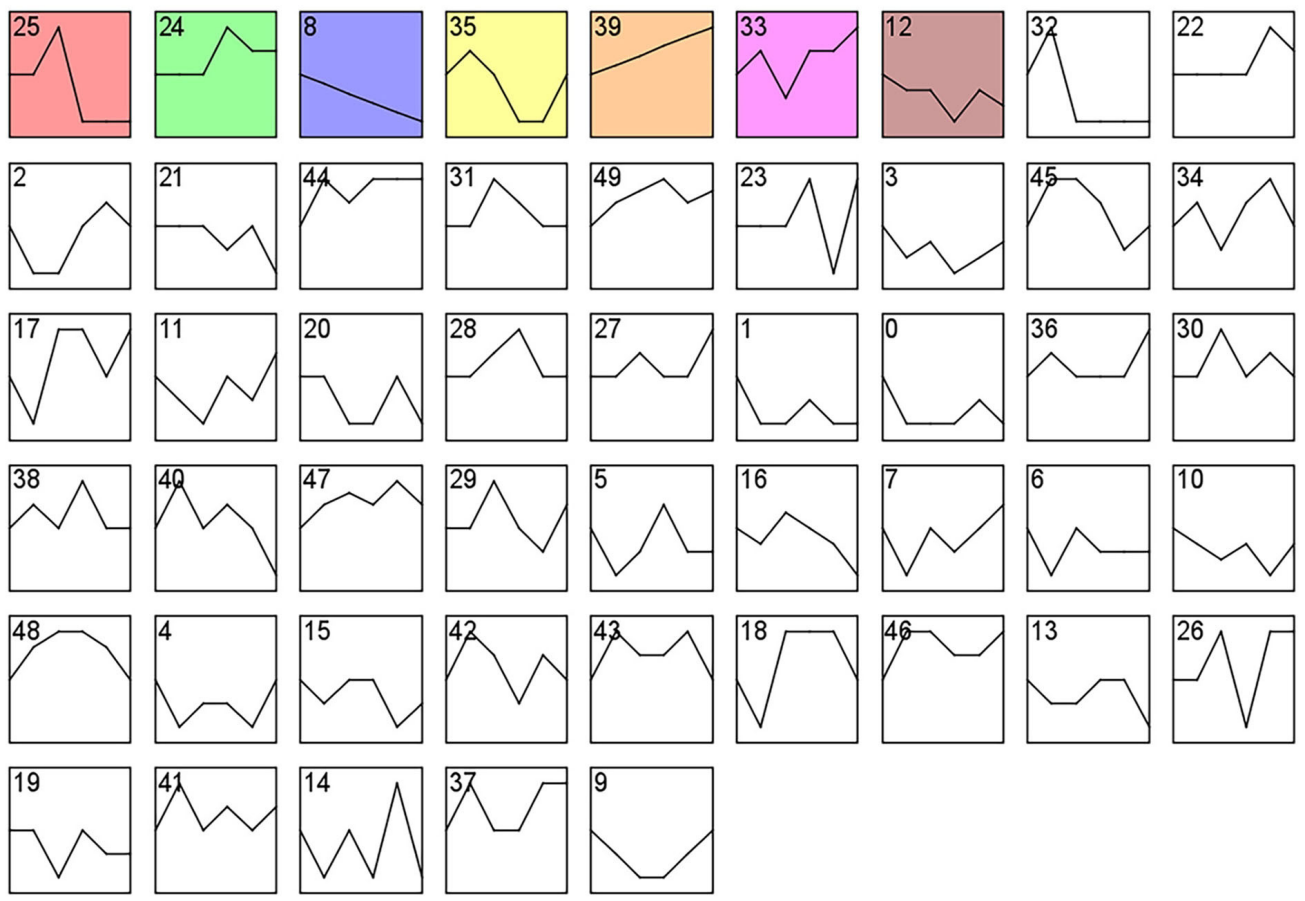
Gene expression profile clusters. Differential gene expression patterns on PHPO axis-related tissues. The vertical axis indicates gene expression levels. Blocks represent differential genes with the same expression pattern. White blocks indicate *P*-values >0.05 and colored blocks indicate *P*-values < 0.05.

Analysis of gene expression profiles using the STEM program allowed identification of potential pathways and DEGs within the PHPO axis that regulate estrus in Tan sheep. A total of 50 modules were obtained from 0 to 49 after the analysis, and these modules could be rearranged according to the number of genes assigned and the significance *P* value (Figure 6, Supplementary Table S6). The top seven colored modules in the top were significantly different (*P* < 0.05), these seven significant modules were 25, 24, 8, 35, 39, 33, and 12, while the remaining white modules were not significantly different (*P* > 0.05). The gene expression trends in the colored modules 25, 24, 8, and 35 were similar, while those in the colored modules 8 and 39 were opposite. As a result, the DEGs discovered from the colored modules 25, 24, 8, and 35 with similar and considerably divergent gene expression trends can be subjected to bioinformatics analysis to further investigate the core genes regulating seasonal reproduction and core genes in Tan sheep (Supplementary Figure S1).

### PPI analysis of the differentially expressed MRNAs

**Figure 7:**
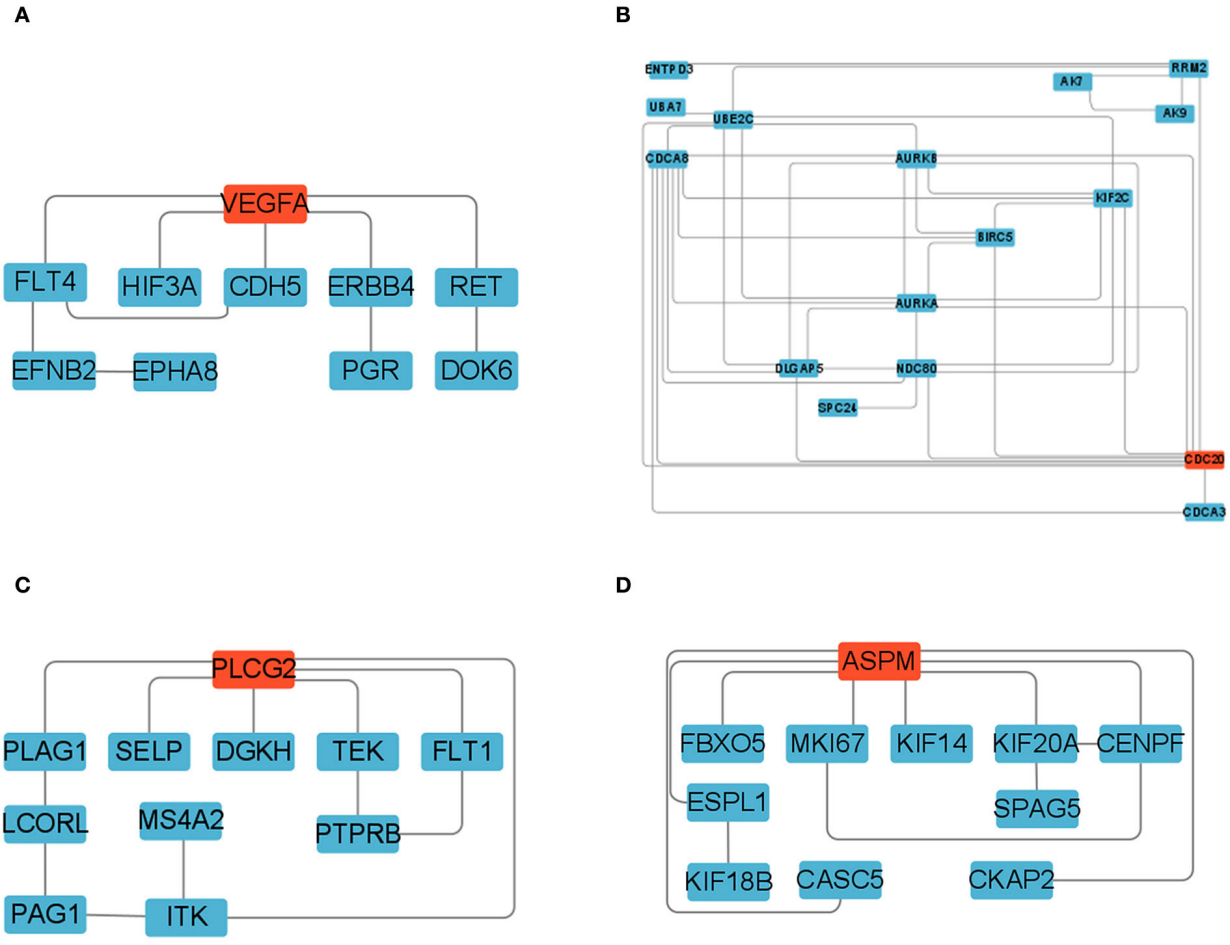
Protein-protein interaction network diagram. **(A–D)** Represent clusters 25, 24, 8, and 35, respectively. Key candidate genes are indicated in red, and differentially expressed genes in the network are indicated in blue.

We constructed four separate protein interaction networks (Figure 7) from the DEGs identified in the colored Cluster (25, 24, 8, 35). The DEGs encoding proteins in the PHPO axis tissue were visualized by PPI to identify regulatory networks within the PHPO axis that may regulate seasonal breeding of Tan sheep (Table 3). Cluster 25 generates a PPI network with 10 nodes and 10 edges, Cluster 24 generates a PPI network with 16 nodes and 41 edges, and Cluster 8 and 35 both generate a PPI network with 11 nodes and 12 edges. Subsequently, the core genes were screened from the top-ranked DEGs by using the Degree analysis method in the CytoHubba plugin. As a result, there core genes including *VEGFA, CDC20, PLCG*2, *ASPM* were identified as key candidate DEGs within the PHPO axis in Cluster 25, 24, 8, and 35. *VEGFA* was found in the pituitary and *VEGFA* is able to regulate the development of blood vessels near the corpus luteum and follicles. *CDC20* was found to inhibit oocyte meiosis in the pituitary gland and ASPM in the ovary, while *ASPM* promoted oocyte meiosis. In addition, *PLCG2* was found in the hypothalamus, and *PLCG2* regulates the development of the uterine muscles and epididymis in animals.

**Table 3:** The key DEGs enriched in PHPO axis-related tissues.

**Tissue**	**Cluster 25**	**Cluster 24**	**Cluster 8**	**Cluster 35**
Pineal	FLT4↑EPHA8↑		DGKH↑	
Hypothalamus	DOK6↑HIF3A↑	SPC24↓	PLCG2↑TEK↑ITK↑	
	RET↑ERBB4↑		FLT1↑SELP↑MS4A2↑	
Pituitary		RRM2↓AURKB↓ UBE2C↓CDCA3↓	PAG1↑LCORL↑	
		AURKA↓DLGAP5↓	PLAG1↑	
		KIF2C↓CDCA8↓ CDC20↓NDC80↓		
		BIRC5↓		
Ovary	CDH5↑EFNB2↑	AK7↓ENTPD3↓	PTPRB↑	ASPM↑CKAP2↑KIF20A↑
	VEGFA↑	AK9↓		FBXO5↑MKI67↑KIF18B↑
				CENPF↑KIF14↑SPAG5↑
				ESPL1↑CASC5↑

“↑” indicates gene up-regulation and “↓” indicates gene down-regulation.

### Validation of transcriptome sequencing results

**Figure 8:**
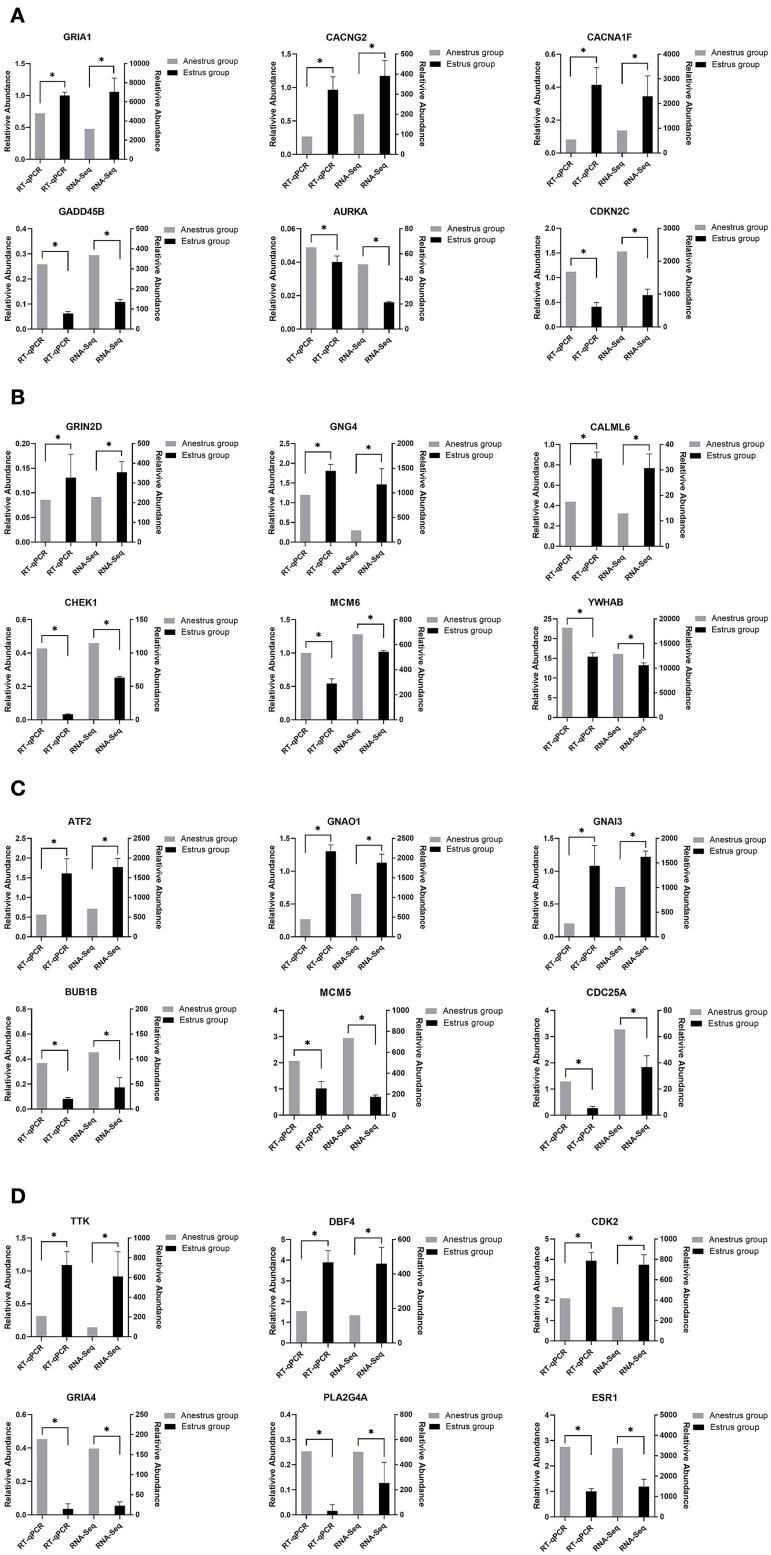
This figure shows the gene expression levels between RNA-Seq and qRT-PCR in four tissues: **(A)** Pineal, **(B)** Hypothalamus, **(C)** Pituitary, and **(D)** Ovary.

To verify the differentially expressed genes identified by transcriptome sequencing, six differentially expressed genes were randomly selected on each of the four tissues of the PHPO axis, and gene expression was confirmed by RT-qPCR using β-actin as an internal reference gene. The results showed consistent gene expression trends between both RT-qPCR and RNA-Seq (Figure 8). Therefore, it indicates that the RNA-Seq results are reliable and can be used for bioinformatics analysis of sequencing results.

## Discussion

It is critical to investigate the seasonal estrus regulating mechanism in Tan sheep in order to improve their reproductive efficiency. The gene regulatory network is a continuous and complicated dynamic system, and gene regulation is a dynamic event that varies with the environment. Researchers can use RNA-Seq to uncover the gene regulatory network of the animal organism (17). The network pathways of gene regulation can be identified by sequencing the transcriptome of tissues or cells under various conditions, which allows biological analysis of the data to look for differential genes and important signaling pathways that are enriched. Transcriptome sequencing can provide insight into the variations in gene expression levels in the pineal, hypothalamus, pituitary, and ovarian tissues of Tan sheep during estrus and anestrus, which can serve as foundation for elucidating the molecular mechanisms of seasonal breeding regulation in Tan sheep.

The length of external light and the hormones generated by the reproductive system have an impact on the reproductive activity of Tan sheep. Previous research on sheep mainly focused on adipose tissue (18) or skin tissue (19) to identify the molecular pathways related with economic features. Another studies also determined some key genes and signaling pathways relating to the reproductive regulation of Tan sheep in pituitary (16) or ovarian (20) in sheep. In contrast, we first performed a combined transcriptome sequencing analysis across the pineal-hypothalamic-pituitary-ovarian axis and identified some hub genes and key signaling pathways regulating seasonal breeding in Tan sheep, this can provide some insight into the molecular regulation mechanism of seasonal reproduction in Tan sheep. We screened 940, 1,638, 750, and 971 DEGs in the pineal, hypothalamus, pituitary and ovary of Tan sheep during estrus and anestrus, respectively. This is the first study that, to our knowledge, examined the variations in gene expression and regulatory networks across the entire PHPO axis by RNA-seq during estrus and anestrus in Tan sheep.

In the pineal gland, hypothalamus, pituitary, and ovarian tissues of Tan sheep, the bile acid secretion pathway was identified as a common signaling pathway by the KEGG pathway analysis. Nuclear receptors (NRs) are a family of transcription factors that include endocrine receptors activated by steroids and thyroid hormones, which regulate target genes though binding its ligands and can play a role in biological processes such as energy metabolism, reproduction, and development (21). FXR, the bile acid nuclear receptor, has been found to be expressed in the mouse testis and to influence reproduction in male mice by regulating the expression of the pluripotency marker Lin28 in germ cells (22). It has also been demonstrated that FXR is also expressed in mouse ovarian granulosa cells and that FXR can regulate the function of granulosa cells by acting on target genes such as SHP and FABP6 (23). Another study discovered that the bile acids can be transported from the blood to the follicular fluid and have an impact on the reproductive system (24). Schuermann et al. (25) used metabolomics to show that metabolites like bile acids are present in the follicular fluid of cows and suggested that these metabolites may be involved in regulating reproductive performance at different physiological stages in cows. Also, studies in humans have found that total bile acid levels in human follicular fluid are twofold higher than in serum, and that bile acid synthesis may compete with steroid hormone synthesis in follicular and granulosa cells (26). Furthermore, total bile acid levels in dominant follicle follicular fluid were higher than in non-pregnant cows, and bile acid transport proteins and receptors were higher in dominant follicle granulosa cells (27). Yang et al. (28) also found differences in bile acid metabolites in follicular fluid between healthy human ovaries and polycystic ovary syndrome, suggesting that bile acid metabolism in follicles may affect follicular development. Similar to the above results, we found a common bile acid secretion pathway in the pineal-hypothalamic-pituitary-ovarian axis between the estrus group and anestrus group in Tan sheep, as well as enrichment of DEGs in the pathway, suggesting that the bile acid secretion pathway is also likely to be involved in relevant reproductive events and may regulate seasonal reproduction across the PHPO axis in Tan sheep.

In addition, we found that the neuroactive ligand-receptor interaction pathway was enriched in three tissues: pineal, hypothalamus and pituitary. The neuroactive ligand-receptor interaction pathway regulates reproductive activity according to transcriptomic studies on ducks (15, 29), chickens (30), and fish (31, 32). Another study found that nutritional restriction affects sow estrus *via* the neuroactive ligand-receptor interaction pathway (33). Similarly, mRNA transcriptome sequencing of the goat hypothalamus revealed that the neuroactive ligand-receptor interaction pathway influences sexual maturation (34). As a result, we propose that the neuroactive ligand-receptor interaction pathway may also be an important in the pineal-hypothalamic-pituitary (PHP) axis of Tan sheep, which may regulate seasonal breeding in Tan sheep.

STEM allows for the analysis of biological data gene expression clustering in a short period of time (35). Pizzi et al. (36) suggested that genes with similar expression have similar biological functions and work in concert. Fan et al. (37) used STEM to examine genes associated with mammary development at peak, mid and late lactation in dairy cows and found that DEGs are mainly involved in apoptosis and energy metabolism, suggesting the reliability of the STEM analysis method. In the present study, we obtained four colored Clusters with the same trend (25, 24, 8, and 35) by STEM analysis of DEGs cross PHPO axis in Tan sheep. PPI analysis was performed on the DEGs contained in the four Clusters, and four key core genes were displayed, namely *VEGFA, CDC20, ASPM*, and *PLCG2*.

We discovered VEGFA in the ovary, which, along with its receptor FLT4, is the central protein in Cluster 25 protein network. The network includes six DEGs encoding proteins of pineal and hypothalamic, and three DEGs encoding proteins of ovarian. The establishment of the peripheral vascular network is tightly linked to the growth and development of associated tissues in animal reproduction (38). *VEGFA*, as one of the VEGF family, is mainly involved in angiogenesis, and *VEGFA* can control the growth of blood vessels close to follicles and corpus luteum in the ovary (39–41). Numerous studies have shown that the development of vasculature in reproduction-related tissues helps to enhance the synthesis and transmission of endocrine factors, promoting follicular development and luteal formation (42, 43). Castle-Miller et al. (44) discovered that melatonin might modulate gonadotropin gene expression during the breeding and non-breeding seasons by regulating VEGF synthesis. Additionally, it was discovered that the localization of VEGFA and its receptor FLT4 within the relevant tissues can alter with different estrous cycles of animals (45, 46). This further suggests that *VEGFA* genes may play an important role in regulating seasonal breeding of animals. In our study, *VEGFA* and the *FLT4* were found to have comparable expression profiles in Cluster 25, and the expression of DEGs was upregulated in the PPI network formed by Cluster 25, indicating that these DEGs may be crucial in the regulation of animal seasonal breeding. In addition, three signal transduction routes are also involved in the VEGF signaling system (Supplementary Figure S2), the first of which is the Ca2^+^ signaling pathway, which controls mesenchymal cell production of steroid hormones as well as cell autophagy, apoptosis, and proliferation (47, 48). Another is the MAPK/extracellular signal-regulated kinase pathway, which is essential for oocyte maturation (49, 50). There is also a phosphatidylinositol 3-kinase/protein kinase signaling pathway that promotes primordial follicle activation and reduces apoptosis in sheep (51, 52). As a result, we hypothesize that there may be a network of VEGFA signal pathway regulating seasonal breeding in the PHO axis of Tan sheep. Of course, more research is needed to uncover the molecular network mechanism of VEGFA regulation of seasonal breeding in Tan sheep.

In addition, we discovered that the DEGs contained in the PPI network formed by Cluster 24 and 35 were primarily located in the pituitary and ovaries. Both CDC20 and ASPM are associated with meiosis, but interestingly, CDC20 inhibits meiosis while ASPM promotes it. CDC20 is a co-activator of the anaphase-promotion complex/cyclosome (APC/C) during mitosis and can maintain the genome by controlling the spindle assembly checkpoint (53). The activation of APC/C by CDC20 is a critical stage in the transition from meiosis I to meiosis II and homolog segregation in oocyte meiosis (54). This shows that CDC20 plays an important role in regulating mammalian follicle development and oocyte maturation. It has been demonstrated that CDC20 is able to maintain oocyte meiosis arrest in pig (55), mice (56), and cattle (57). ASPM is found in the mammalian cell intermediate (58) and is localized at both poles of the spindle during cell mitosis (59). According to the research, ASPM is essential for meiotic spindle assembly and meiotic progression in mouse oocytes (60). Additionally, it has also been found that estrogen has a conserved depressed effect on *ASPM* expression in primates (61). Therefore, we speculate that it is possible that CDC20 and ASPM may form e a dynamic balance in the oocyte division cycle, and hence may play an important role in seasonal reproduction of Tan sheep by regulating follicle development.

In Cluster 8, *PLCG2* (the gene encoding phospholipase Cγ2) was found to be located at the core of the PPI network. It was found that *PLCG2* may be involved in the regulation of placental function in goats (62). Additionally, *PLCG2* has also been discovered to control uterine muscle activity in laying hens (63). Meanwhile, Környei et al. (64) discovered that *PLCG2* is also expressed in human myometrium, which may be involved in the regulation of myometrial cell differentiation, maturation and uterine tissue growth. In addition, it was also demonstrated that *PLCG2* can regulate the development of epididymis in mice, and that male mice lacking *PLCG2* are sterile (65). Accordingly, we supposed that *PLCG2* may affect animal reproduction by regulating the growth and development of animal reproductive organs. In present study, we discovered that the DEGs in the PPI network, which consisted of Cluster 8 with *PLCG2* as the core gene, were all upregulated between the two controls and were dispersed cross all four tissues. Therefore, we speculate that *PLCG2* may similarly play a role controlling seasonal reproduction of Tan sheep along the PHPO axis, however this remains to be proven in future studies.

## Conclusion

In this study, RNA-Seq was used to identify genes and signaling pathways associated with the regulation of seasonal reproduction in the PHPO axis of Tan sheep. We discovered that two pathways, bile acid secretion and neuroactive ligand-receptor interaction, may be related to the regulation of seasonal reproduction in Tan sheep. We also identified four core genes, *VEGFA, CDC20, ASPM*, and *PLCG2*, which are closely associated with the regulation of seasonal reproduction in Tan sheep. Our findings can provide the foundation for further research in the molecular regulation mechanisms of seasonal reproduction, as well as certain references for molecular breeding of high-fertility in Tan sheep. Although we have discovered core genes and key signaling pathways that regulate seasonal reproduction throughout the PHPO axis, we still do not fully understand the specific molecular mechanisms by which these key genes and the key signaling pathways regulating seasonal reproduction in Tan sheep. Therefore, it is necessary to conduct functional and mechanistic studies on these genes and signaling pathways in the future.

## Data availability statement

The original contributions presented in the study are included in the article/Supplementary material, further inquiries can be directed to the corresponding author/s.

## Ethics statement

All experimental animal studies were reviewed and approved by the Animal Welfare Committee of Ningxia University (permit number NXUC20180306).

## Author contributions

SW analyzed the data and wrote the paper. XK, CY, FW, and TD performed the experiment, provided text and data for the methods, and contributed comments on the manuscript. XG, ZM, and CL participated in analysis of the data and writing of the manuscript. HZ designed and supervised the experiment. XD supervised the project. All authors read and approved the final manuscript.

## Funding

This research was funded by the National Natural Science Foundation of China (31760665) and the Ningxia Natural Science Foundation (2022AAC03057).

## Conflict of interest

The authors declare that the research was conducted in the absence of any commercial or financial relationships that could be construed as a potential conflict of interest.

## Publisher's note

All claims expressed in this article are solely those of the authors and do not necessarily represent those of their affiliated organizations, or those of the publisher, the editors and the reviewers. Any product that may be evaluated in this article, or claim that may be made by its manufacturer, is not guaranteed or endorsed by the publisher.
