# Supplementary material for: Analysis of reproduction-related transcriptomes on pineal-hypothalamic-pituitary-ovarian tissues during estrus and anestrus in Tan sheep
Source: Front Vet Sci. 2022 Nov 24;9:1068882. doi: 10.3389/fvets.2022.1068882 (PMC9729709; doi:10.3389/fvets.2022.1068882)
Supplement: Supplementary file 1 [file Table_1.DOCX]

Table 1 Pineal real-time fluorescence quantification primer sequence

| Gene | Primer sequence(5'-3') | Annealing temperature/℃ |
| --- | --- | --- |
| GADD45B | F:CCATCGACGAGGAAGAGGAGGAC R:ATGTCGTTGTCGCAGCAGAAGG | 60.0 |
| CDKN2C | F:CCGTGGTGGAGTTCCTTGTGAAG R:ACGACCTCGTTTCTCCGGTAGAG | 60.0 |
| AURKA | F:AATTCCTCGCAGCGTGTTCCTTC R:TTGCTCTTCTGGGTGTTACTTGGTG | 60.0 |
| GRIA1 | F:TCATCTCCTCCTACACAGCCAACC R:GTCCCGTAAGCGATTTCCGTCTG | 60.0 |
| CACNA1F | F:CATCCTTGCTGCTTCTCCTCTTCC R:TTGGTGTGGGTCTGGTCAAAGTTG | 60.0 |
| CACNG2 | F:TGGGAACCGACTATTGGCTCTACTC R:GGCAGCAGGTTCTCCATAATCCAG | 60.0 |
| β-actin | F:GGCATTCACGAAACTACCTTC  R:ATCTCTTTCTGCATCCTGTCTG | 60.0 |

Table 2 Hypothalamic real-time fluorescence quantitative primer sequence

| Gene | Primer sequence(5'-3') | Annealing temperature/℃ | |
| --- | --- | --- | --- |
| CHEK1 | F:CGGCTTTCTAAGGGTGATGGATTGG R:AACCTTCTGGCTGCTCACAACATC | | 60.0 |
| YWHAB | F:AGGAATGAGAAGAAGCAGCAGATGG R:TCCAGCACGTCATTGCAGATGTC | | 60.0 |
| MCM6 | F:AGATGGATGTGCGGGATCAAGTTG R:GGATGTCCTGGCATTCAGAGTAGC | | 60.0 |
| GNG4 | F:AATAACAGCACCGCCAGCATCTC R:CCGCACATGAGCTTCACAGTAGG | | 60.0 |
| GRIN2D | F:CTCTCGGGAATCTGCCACAATGAC R:GCCACCAGGAGCATGTAGAAGAC | | 60.0 |
| CALML6 | F:GGCGAGCTGAGAACAGTCTTGTG R:ATTCCCGTTGGCTTCCTTCATCATC | | 60.0 |
| β-actin | F:GGCATTCACGAAACTACCTTC  R:ATCTCTTTCTGCATCCTGTCTG | | 60.0 |

Table 3 Pituitary real-time fluorescence quantitative primer sequence

| Gene | Primer sequence(5'-3') | Annealing temperature/℃ | |
| --- | --- | --- | --- |
| CDC25A | F:GCACATGGAAGAGGAGGTTGAAGAG R:ACACCACGATAACACGCTTGCC | | 60.0 |
| BUB1B | F:AGAGACAAGCCCAGACCCAGTG R:TCTGCCTCGCTCATCAGTACGG | | 60.0 |
| MCM5 | F:CAAGGATGAGCACAATGAGGAGAGG R:GCGGCAGTAGGCGATGAACTTC | | 60.0 |
| GNAO1 | F:GTACGGTGACAAGGAGCGAAAGG R:GACCTGTTGAAGCACTCCTGGATC | | 60.0 |
| GNAI3 | F:CGGTGCTGGAGAATCTGGCAAAAG R:ATGGCCCTTATGATTGCGATGATGG | | 60.0 |
| ATF2 | F:AACGGCGATACTGTCAAAGGTCAC R:TGGATGTGGCTGGCTGTTGTAATG | | 60.0 |
| β-actin | F:GGCATTCACGAAACTACCTTC  R:ATCTCTTTCTGCATCCTGTCTG | | 60.0 |

Table 4 Ovarian real-time fluorescence quantitative primer sequence

| Gene | Primer sequence(5'-3') | Annealing temperature/℃ |
| --- | --- | --- |
| ESR1 | F:CTGCTGCTGGAGATGCTGGATG R:GCTGGCTCTGATTCACGTCTTCC | 60.0 |
| PLA2G4F | F:ACTTCACCTACGAGCCCCAAGAG R:CCTTCCGCACAGTCTCCACATTG | 60.0 |
| GRIA4 | F:AGCGTGCAAATAGGTGGTCTCTTC R:GTTGGCGGTCTCGATGTTGTCC | 60.0 |
| PLA2G4A | F:CGAGAAGGACTGAAGGAATGCTACG R:CTCTTGGAACACCTGGAGCCTTG | 60.0 |
| CDK2 | F:GCACTCACTGGCATTCCTCTTCC R:ATGGACCCATCTGCGTTGATAAGC | 60.0 |
| TTK | F:CATTCCTGAGCTTCTGGCACATCC  R:TTCATTTCTTCAGCGGCTCCCTTAG | 60.0 |
| β-actin | F:GGCATTCACGAAACTACCTTC  R:ATCTCTTTCTGCATCCTGTCTG | 60.0 |
