# Supplementary material for: Analysis of reproduction-related transcriptomes on pineal-hypothalamic-pituitary-ovarian tissues during estrus and anestrus in Tan sheep
Source: Front Vet Sci. 2022 Nov 24;9:1068882. doi: 10.3389/fvets.2022.1068882 (PMC9729709; doi:10.3389/fvets.2022.1068882)
Supplement: Supplementary file 7 [file Data_Sheet_1.PDF]

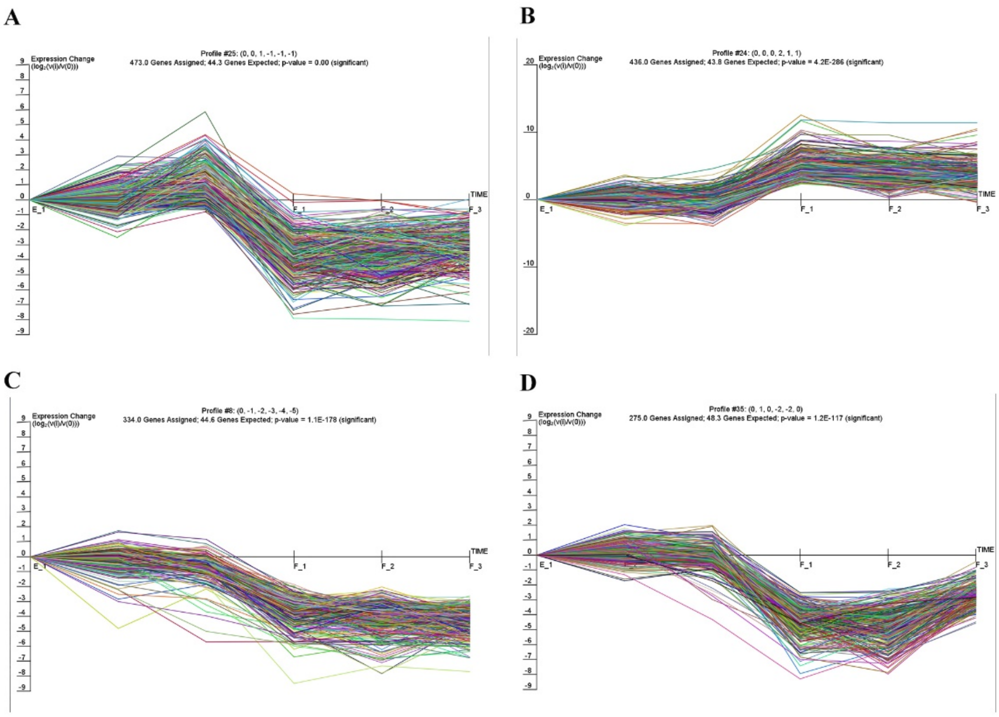

Supplementary Figure S1. Gene expression profile. Figure A, B, C and D represent the trend of differentially expressed genes contained in cluster25, 24, 8 and 35, respectively.
