# Supplementary material for: Analysis of reproduction-related transcriptomes on pineal-hypothalamic-pituitary-ovarian tissues during estrus and anestrus in Tan sheep
Source: Front Vet Sci. 2022 Nov 24;9:1068882. doi: 10.3389/fvets.2022.1068882 (PMC9729709; doi:10.3389/fvets.2022.1068882)
Supplement: Supplementary file 8 [file Data_Sheet_2.PDF]

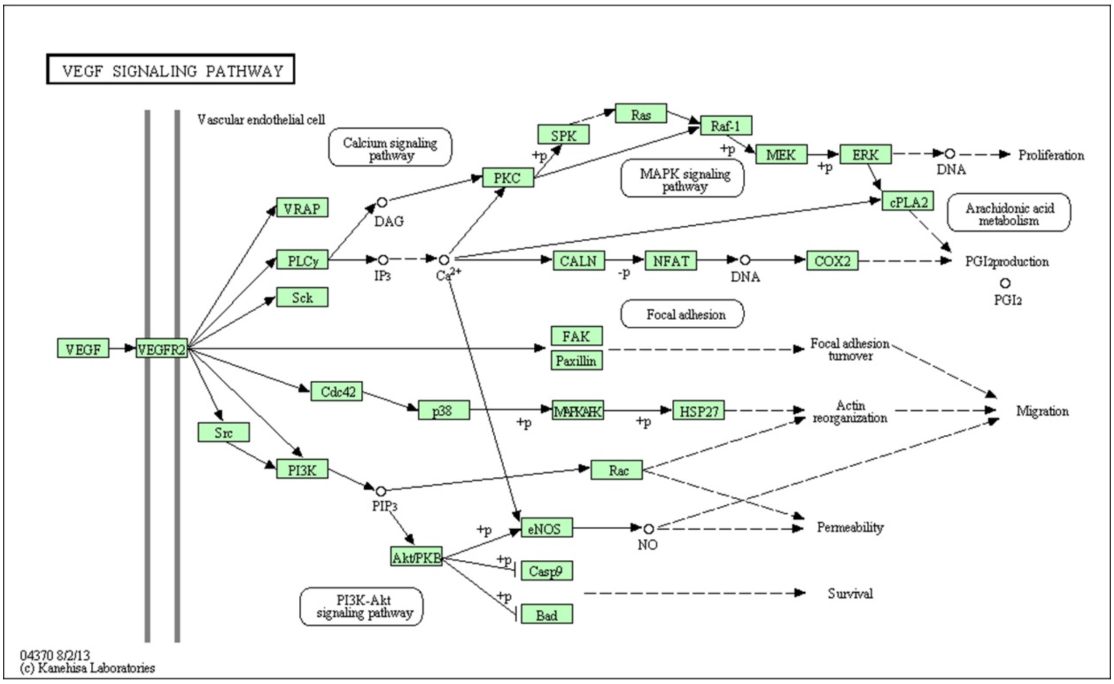


Supplementary Figure S2.VEGF signaling pathway diagram,This pathway also contains three signal transduction pathways, calcium, MAPK, and PI3K/AKT.
